# Prevalence and clinical characteristics of patients with obsessive-compulsive disorder in first-episode psychosis

**DOI:** 10.1186/1471-244X-13-156

**Published:** 2013-05-30

**Authors:** Kristen Hagen, Bjarne Hansen, Inge Joa, Tor Ketil Larsen

**Affiliations:** 1 Østmarka Psychiatric Department, St. Olav’s University Hospital, Trondheim, 7441, Norway; 2 NTNU Norwegian University of Science and Technology, Hogskoleringen, Trondheim, 1, 7491, Norway; 3 Department of Psychiatry, Haukeland University Hospital, Bergen, Norway; 4 Department of Neuroscience, Norwegian University of Science and Technology, Trondheim, Norway; 5 Psychiatric Division, Regional Centre for Clinical Research in Psychosis, Stavanger University Hospital, Stavanger, Norway; 6 University of Stavanger, Faculty of Social Sciences, Stavanger, Norway

**Keywords:** Obsessive-compulsive disorder, First-episode psychosis, Schizophrenia, Prevalence, Comorbidity

## Abstract

**Background:**

Obsessive-compulsive disorder (OCD) in patients with psychotic disorders has been reported to be a frequent co-morbid disorder in patients with psychotic disorders. The aim of the study determine the prevalence of OCD in first-episode psychosis and the relationship with clinical characteristics.

**Methods:**

First-episode psychosis patients (N = 246) consecutively admitted to a comprehensive early psychosis program were assessed for OCD with the Structured Clinical Interview for DSM-IV. Symptom assessment measures were the Positive and Negative Syndrome Scale, Global Assessment of Functioning, and the Clinician Rating Scale.

**Results:**

Twenty-six patients (10.6%) fulfilled the criteria for OCD. Patients with comorbid OCD were younger, had more depressive symptoms and a higher rate of suicidal plans or attempts at index point compared to patients without OCD. The two groups did not differ with respect to other demographic variables or severity of psychotic symptoms.

**Conclusion:**

OCD is a significant comorbid disorder in patients with first-episode psychosis. Since treatment procedures are different, systematic screening for OCD is warranted.

## Background

The existence of comorbid OCD in patients with psychotic disorders has been recognised for over a century, but the reports have been less consistent with regard to the extent of the comorbidity and the influence on the clinical expression. Comorbidity rates are in general reported to be higher than the prevalence in the general population. In a recent meta-study Achim et al. estimated comorbidity rates for OCD in patients with schizophrenia at 12% [1].

Several studies have proposed that comorbid OCD affect the clinical expression of patients with psychotic disorders. Comorbid OCD is related to more impairmed social functioning [2,3] and more neurological abnormalies [4-6]. Patients with schizophrenia and comorbid OCD have been reported to have earlier onset of psychosis, and an increased rate of depressive symptoms and suicidal attempts [7-9], compared to their non-OCD counterparts. However, these patterns have not been constantly reported [10,11].

Most studies of comorbid OCD in psychotic disorders have been conducted on patients with schizophrenia in large psychiatric institutions or day treatment centres. The samples often consist of chronic patients, which introduce several limitations related to the effects of illness duration, institutionalisation, and aggravation or induction of second-onset OCD during antiserotonergic antipsychotics [12-14]. To increase the understanding of comorbid OCD in psychosis, there is a need for studies where these methodological challenges are met.

The present report is from a representative and relatively large sample of patients with first-episode psychosis (FEP), recruited in a population-based catchment area in an early phase of the psychotic disorder. The primary aim was to investigate the prevalence of OCD in the FEP-patients, with strict diagnostic methodology. The secondary aim was to investigate whether the patients with comorbid OCD differed from the patients without OCD on socio-demographic characteristics, type of psychotic disorder, general functioning, and severity of positive and negative symptoms at the start of treatment.

## Method

### Participants

This study included patients in the Catchment area for Stavanger University Hospital, division of psychiatry in the South sector of Rogaland County, Norway, with a population of about 270 000, mainly in urban and suburban areas. Recruitment for the study continued consecutively from January 1, 2002 to November 30th, 2010. The criteria for inclusion in the study were 1) Living in the catchment area; 2) age 15–65 years; 3) meeting the DSM-IV [15] criteria for first episode schizophrenia, schizophreniform psychosis, schizoaffective psychosis, delusional disorder, brief psychosis, affective disorder with mood incongruent delusions, or psychosis not otherwise specified, and also from August 1, 2008 Substance induced psychosis; 4) being actively psychotic, as measured by a score at the Positive and Negative Syndrome Scale (PANSS) [16] of four or more on at least one of the following PANSS items; P1 (delusions), P3 (hallucinations), P5 (grandiose thinking), P6 (suspiciousness) and A9 (unusual thought content); 5) not previously receiving adequate treatment for psychosis (defined as antipsychotic medication of 3.5 haloperidol equivalents for 12 weeks or until remission of the psychotic symptoms); 6) no neurological or endocrine disorders with relationship to the psychosis; 7) no contraindications to antipsychotic medication; 8) understands &frasl; speaks Norwegian; 9) IQ over 70 measured by the Wechsler intelligence scales for adults (WAIS) [17]; 10) willing and able to give informed consent.

The present study is part of the Early Treatment and Intervention in Psychosis Study-2 (TIPS-2). This is a continuation of the TIPS study, which was an early detection program that consisted of repeated information campaigns directed at the general population, schools, and first-line health care personnel and low threshold detection clinical teams capable of rapid assessment and triage [18]. The study was approved by the Regional Committee for Medical Research Ethics Health Region West (015.03). Written informed consent was obtained from all study participants. Parents or legal guardians gave informed consent for patients younger than 18 years of age. The patients entered the study through the early TIPS-2 study’s low-threshold detection team, or were referred to the hospital acute inpatient ward or the outpatient clinics by local general practitioners or psychiatrists. After a preclinical screening interview, a senior psychiatrist or psychologist examined the patients. Demographics and supplementary information were collected, and the SCID-I interview was conducted.

Altogether, 481 consecutive patients were identified. Seventy patients were excluded (21 were not registered in the catchment area, 12 because of poor language skills, 11 because they were younger than 15 years of age, and 6 because of low IQ; 20 patients were lost for further study contact and were not asked). Of the 411 remaining patients, 166 refused participation. The rate of consent to participate was therefore 60% (246 patients). This report comprises data from the time of inclusion. All patients were assessed within a week of contact and assigned to the standard treatment program, which consisted of an antipsychotic medication algorithm, multifamily work, and active outreach-supportive psychotherapy [19].

### Assessments

Assessment teams consisting of clinically experienced and trained research personnel carried out all of the assessments. The structured clinical interview for the DSM-IV (SCID-I) [20] was used for diagnostic purposes. Symptom levels were measured by the Positive and Negative Syndrome Scale score (PANSS) [16]. Symptom domains are represented by the corresponding PANSS components (positive, negative, excitement, depressive and cognitive). Global functioning was measured by the Global Assessment of Functioning Scale (GAF) [15]. The scores were split into symptom [GAFs] and function [GAFf] scores to improve psychometric properties [21]. Drug and alcohol misuse was measured by the Clinicians Rating Scale [22]. The duration of untreated psychosis (DUP) was measured retrospectively, based on all of the available sources. The DUP is reported as weeks from onset of psychosis (the first week with symptoms corresponding to a PANSS score of 4 on P1, 3, 5, 6 or G9) until the start of adequate treatment [18,23].

Suicidal behavior was assessed at the index diagnostic interview, by asking the patients whether they had experienced any suicidal thoughts, plans, or attempts both in the preceding month and earlier in their lifetime. The highest reported level of suicidal behavior for the time period was registered [24]. The variable was dichotomised into severe suicidality (present or not), where severe suicidality was defined as plans or attempts [23], based on previously shown high associations between plans, attempts and later completed suicides [25].

The assessors were all previously trained to reliabel use of the instruments. Reliability of measurements were calculated and ranged from fair to very good (DUP, 0.99; GAFs score, 0.63; GAFf score, 0.88; PANSS positive sum score, 0.88; PANSS negative sum score, 0.76; and PANSS general sum score, 0.56 [all intra-class correlations, 1.1]; for diagnostic categories, K = 0.76) [19].

### Diagnostics of OCD

The diagnostics of OCD were based on a stringent three-step procedure. The first step was the original SCID-I interview, which was accomplished by members of the detection team trained for reliability (for details see earlier publications; [26,27]). The second step was an independent review in which the SCID-I and PANSS reports which were a part of each patient’s file, and the patients’ full medical records were re-assessed. The first author, a psychologist with extensive training in OCD diagnostics, performed the reviews. In the third step, all cases in which obsessions or compulsions were mentioned in either PANSS, SCID-I, or in the medical record, were re-examined jointly by a senior psychiatrist (T.K.L) and a senior psychologist (B.H), both with extensive experience in diagnostics of OCD and psychosis. The medical records were electronically stored and we thus had the ability to search each one for relevant concepts, such as *obsessive*, *compulsive*, *ritual*, *exposure therapy*, and *SSRI*. The diagnostics were based on a strict definition of OCD, and the obsessive-compulsive symptoms had to be unrelated to the psychotic content.

### Statistics

Continuous data were presented as means with standard deviations (SD) and analysed using Students t-test. Categorical variables were analysed using Chi-square. A five-factor PANSS component analysis was used to investigate divergences between OCD and non-OCD in dimensions in the PANSS interview [28]. Significance level was set at P < 0.05. Analyses were performed using SPSS 18.0.

## Results

### Prevalence of OCD in FEP-patients

Of the 246 participants, 16 patients (6.5%) were originally diagnosed with OCD, and 10 more cases were diagnosed with OCD after the reassessment of the OCD diagnostics. All of the patients initially diagnosed with OCD were diagnosed with OCD at the OCD-reassessment. Altogether, 26 patients (10.6%) were found to fulfil the DSM-IV criteria for OCD.

### Comparison of demographic characteristics between OCD and non-OCD FEP-patients

Table 1 summarises demographic characteristics between OCD and non-OCD FEP patients. The OCD patients were younger at start of treatment than their non-OCD counterparts. There were no other significant differences between the groups.

### Comparison of diagnostics between OCD and non- OCD FEP- patients

Among the patients with schizophrenia spectrum disorders, five patients (5.4%) were diagnosed with co-morbid OCD. This is a significantly lower prevalence than for the non-schizophrenia spectrum disorders. None of the patients with schizophrenia were diagnosed with OCD. Affective psychosis and psychosis-NOS were the diagnoses with highest prevalence of comorbid OCD (Table 2).

### Comparison of clinical symptoms and function

The OCD-patients had more depressive symptoms on the PANSS, they also had more suicidal plans and attempts during the last month before hospitalisation. There was, however no difference in reported lifetime prevalence of suicidal plans or attempts. There were no significant differences between the two groups on the other demographically characteristics (Table 3).

## Discussion

In this study we found the prevalence rate of comorbid OCD among patients with FEP to be 10.6%. The reported prevalence is higher than the prevalence of OCD in FEP in studies by Craig et al. [13], Sim et al. [29], and Sterk et al. [30]. The independent reassessment of the OCD-diagnostics raised the prevalence from 6.5% to 10.6%; this was a procedure that, to our knowledge, was not presented in other studies of OCD in FEP-patients. Our sample included affective psychosis with mood-incongruent delusions in the FEP-sample. Since this was the diagnostic group with the highest number of OCD-patients in the sample, the inclusion of this patient group in the sample may have raised the OCD-prevalence.

Our study indicated a significantly lower comorbidity of OCD in patients with schizophrenia spectrum disorders. Most noteworthy is the finding that among the patients diagnosed with schizophrenia as their primary diagnosis, none were diagnosed with comorbid OCD. The prevalence of OCD for patients diagnosed with schizophrenia spectrum disorders was in line with the studies from Craig et al. [13], Sterk et al. [30] and Sim et al. [29], but was

**Table 1:** Baseline comparison of demographic characteristics of OCD and non-OCD first episode psychosis

**Variable**	**OCD (N=26)**	**Non-OCD (N=220)**	**P-value**
	**Mean**	**Mean**	
Age (SD)	22.96 (8,007)	27.05 (10,321)	.053*
DUP in weeks mean (SD)/median	61.3(113.9)/14	80.5 (204.9)/20	.497
Male (%)	43.2	58.6	.112
Single marital status (%)	87.5	74.5	.116
Drug abuse %	15.4	29.8	.122
Years of education mean (SD)	11.4 (1.7)	11.8 (2.6)	.501

DUP = duration of untreated psychosis.

* = Significant at .05 level.

**Table 2:** Comparison of diagnostics between OCD and non-OCD in first episode psychosis

**Diagnostics**	**OCD (N=26) N (%)**	**Non-OCD (N=220) N (%)**	**P-value**
Schizophrenia spectrum disorders	5 (19)	87 (40)	.043*
Schizophrenia	0 (0)	52 (24)	
Schizophreniform disorder	2 (8)	10 (5)	
Schizoaffective disorder	3 (12)	25 (11)	
Brief psychosis	0	13 (6)	
Delusional disorder	2 (8)	19 (9)	
Affective psychosis MID	10 (38)	45 (20)	
Psychosis NOS	8 (31)	34 (15)	
Drug induced psychotic disorders	1 (4)	19 (9)	
Psychotic disorder NOS	0	3 (1)	

* = Significant at .05 level.

 lower than the prevalence reported in a study of first-episode schizophrenia spectrum disorders by Poyurovsky and colleagues [12] and in a study of first episode schizophrenia and related disorders by de Haan [31]. In our study, OCD was most prevalent among patients with affective psychosis and psychosis NOS.

The second purpose of the study was to compare demographic and clinical characteristics between the OCD and the non-OCD group. The FEP-patients with OCD were found to have a significantly younger age at the start of treatment than their non-OCD counterparts. This

**Table 3:** Comparison of clinical characteristics of OCD and non-OCD in first episode psychosis

**Variable**	**OCD (N=26)**	**Non-OCD (N=220)**	**P-value**
	**Mean**	**Mean**	
GAF Symptoms (SD)	33.1 (5.7)	31.2 (7.4)	.138
GAF Function (SD)	40.8 (10.4)	39.5 (9.5)	.524
PANSS Total	65.4 (14.1)	64.61 (13.8)	.699
Positive component (SD)	14.5 (3.9)	14.5 (3.9)	.898
Negative component (SD)	19.7 (8.3)	18.7 (7.3)	.542
Excitement component (SD)	8.9 (2.7)	7.9 (3.3)	.174
Depressive component (SD)	14.7 (3.1)	12.3 (3.6)	.002**
Cognitive symptoms (SD)	5.5 (2.8)	5.8 (2.9)	.634
Suicidal behaviour; plans or attempts (index) (%)	26.9	9.6	.009**
Suicidal behaviour; plans or attempts (lifetime) (%)	34.6	27.3	.431|

PANSS = Positive and negative symptom scale; GAF = Global Assessment of Functioning.

**= Significant at .01 level.

 is in line with the findings from Craig and colleagues [13], who reported that a significantly larger portions of the FEP-patients with obsessive compulsive symptoms (OCS) was younger than 21 years compared to FEP- patients without comorbid OCS. Poyurovsky and colleagues [32] reported that adolescent schizophrenia patients with OCD had an earlier onset of psychosis compared with their non-OCD counterparts, and Üçok and colleagues [7,11] reported that among schizophrenia patients, comorbid OCD was related to earlier debut of schizophrenia. No age difference in debut of psychosis were found between OCD and non-OCD patients in hospitalised first-episode schizophrenia patients [12] or in a sample of patients with first-episode schizophrenia and another related disorder [10]. A possible earlier age of debut, however, is noteworthy since it may indicate an accentuated neuro-developmental origin of OCD in patients with psychosis [32].

Patients with co-morbid OCD reported a higher rate of suicidal plans or attempts at index point. This finding is in line with the findings from Üçok et al. [7] and Sevincok et al. [8]. However, this pattern was not reported in a more recent study by Üçok et al. [11]. A heightened rate of suicidal plans or attempts in the OCD-group is an important finding, especially given that previous studies have indicated that suicidality at index point is a predictor for continued suicidality in patients with FEP [23].

The patients with co-morbid OCD scored higher on the depression component of the PANSS. This in line with de Haan et al. [31], who reported that, among patients with recent onset schizophrenia and related disorders, patients with co-morbid OCD were more depressed, as measured by the Montgomery-Asberg Depression Rating Scale (MADRS). The increased prevalence of depressive symptoms and suicidal plans and attempts may be a result of living with OCD, which is supported by findings from Rasmussen & Tsuang [33], who reported that up to 75% of patients with an OCD diagnosis also fulfilled the criteria of a depressive disorder. A possible confounding variable may however be the higher number of patients with affective psychosis among the patients with comorbid OCD. Fontenelle et al. [9] argued that depression may serve as a vulnerability marker for OCD in psychosis since patients with OCD and psychosis reported a higher rate of depressive symptoms both before and after conversion from ultra-high risk for psychosis. The increased depressive symptoms in patients with co-morbid OCD may be clinically important, given findings that people with psychotic disorders and concurrent depressive symptoms have poorer long-term functional outcomes compared to the non-depressed [34], and depressive symptoms have been found to be related to related to an increased risk of suicidality in FEP-patients [35].

In our study, we found no differences between the OCD patients and their non-OCD counterparts in total PANSS score, or in components other than the depressive factors on the five component PANSS analysis [28]. This findings are in line with the results from de Haan and colleagues [10], who did not find any significant relationship between the presence of OCD or OCS and psychotic symptoms in a sample of patients with first episode schizophrenia and related disorders. In a recent meta-study of Cunill et al. [36] they did not report significant differences in PANSS scores between schizophrenia patients with and without OCD. The results from our study may support the hypothesis from Berman and colleagues [37] that obsessive-compulsive symptoms are independent of psychotic symptoms.

It is difficult to compare studies since the prevalence of OCD varies significantly, and many studies have selected samples. A common bias in these estimates of comorbidity has also been the enrolment of patients with OCS and not strictly OCD. This has resulted in a gross overestimaton of co-morbid OCD in many studies [38]. The diagnostics of OCD in patients in our study were based on strict evaluation of the criteria for OCD, based on the guidelines suggested by Bottas and colleagues [39]. The diagnostics were made in three independent steps, to ensure the quality of the assessment. The reassessment of the diagnostics increased the number of OCD-cases from 16 to 26. Interestingly, the re-examination did not reveal any false-positive OCD cases, which may indicate that false negative results may be a more problematic issue in diagnostics of OCD based on SCID-I in FEP-patients than false positive. These findings indicate that an increased focus on OCD in the diagnostics of FEP-patients is clinically important. This may reduce the rate of false negatives, as the diagnostics with SCID-I may underestimate the prevalence of OCD in FEP-patients, even when well-trained assessors carry out the interview. Correct diagnostics are central for optimal treatment [29,38,40].

The study has several strengths. First, the sample is large and from a well-defined catchment area. We assume that nearly every first episode case has been identified and we regard our sample as being highly representative. Second, we independently reassessed all the patients for OCD to ensure the validity of the OCD-diagnostics.

A limitation of the study is that since the primary focus of the parent study was psychosis, there were no assessment instruments specific to OCD included, such as the Yale-Brown Obsessive Compulsive Scale [41,42]. Another limitation is that depressive symptoms were quantified with a second-order method, the PANSS subscale. A third limitation is lack of information about prior treatment (SSRI/CBT) for the patients. It should also be noted that 40% of eligible patients declined to participate, so it is unknown what their diagnoses were and therefore to what extent this may affect the representative nature of the sample.

## Conclusions

OCD co-occurs with psychotic disorders at a higher rate than in the general population. The high co-morbidity rate of OCD reported in patients with psychosis seems not to be a result of chronic illness, antipsychotic medication, or institutionalisation, as we evaluated the prevalence of OCD in a group of FEP-patients with consecutive admissions to a comprehensive early psychosis program.

FEP-patients with OCD tended to have affective disorder and psychosis NOS, were younger, had more depressive symptoms, and reported higher rates of suicidal plans or attempts at the index point. These factors indicate that it is important to detect OCD in FEP-patients. Proper treatment might prevent poor outcomes [43].

## Competing interests

The authors declare that they have no conflict of interest.

## Authors’ contributions

KH, BH, IJ, and TKL contributed to the study design. KH, BH, IJ, and TKL contributed to data collection. KH and IJ conducted the statistical analysis. KH, BH, IJ, and TKL interpreted the data and drafted the manuscript. All authors participated in critical revision of manuscript drafts and approved the final version.

## Pre-publication history

The pre-publication history for this paper can be accessed here:

http://www.biomedcentral.com/1471-244X/13/156/prepub
